# Distinct Serotypes of Streptococcal M Proteins Mediate Fibrinogen-Dependent Platelet Activation and Proinflammatory Effects

**DOI:** 10.1128/iai.00462-21

**Published:** 2022-02-17

**Authors:** Frida Palm, Sounak Chowdhury, Sara Wettemark, Johan Malmström, Lotta Happonen, Oonagh Shannon

**Affiliations:** a Division of Infection Medicine, Department of Clinical Sciences, Lund University, Lund, Sweden; University of Illinois at Chicago

**Keywords:** M protein, *Streptococcus pyogenes*, fibrinogen, platelets, sepsis

## Abstract

Sepsis is a life-threatening complication of infection that is characterized by a dysregulated inflammatory state and disturbed hemostasis. Platelets are the main regulators of hemostasis, and they also respond to inflammation. The human pathogen Streptococcus pyogenes can cause local infection that may progress to sepsis. There are more than 200 serotypes of S. pyogenes defined according to sequence variations in the M protein. The M1 serotype is among 10 serotypes that are predominant in invasive infection. M1 protein can be released from the surface and has previously been shown to generate platelet, neutrophil, and monocyte activation. The platelet-dependent proinflammatory effects of other serotypes of M protein associated with invasive infection (M3, M5, M28, M49, and M89) are now investigated using a combination of multiparameter flow cytometry, enzyme-linked immunosorbent assay (ELISA), aggregometry, and quantitative mass spectrometry. We demonstrate that only M1, M3, and M5 protein serotypes can bind fibrinogen in plasma and mediate fibrinogen- and IgG-dependent platelet activation and aggregation, release of granule proteins, upregulation of CD62P to the platelet surface, and complex formation with neutrophils and monocytes. Neutrophil and monocyte activation, determined as upregulation of surface CD11b, is also mediated by M1, M3, and M5 protein serotypes, while M28, M49, and M89 proteins failed to mediate activation of platelets or leukocytes. Collectively, our findings reveal novel aspects of the immunomodulatory role of fibrinogen acquisition and platelet activation during streptococcal infections.

## INTRODUCTION

Sepsis is a major global public health challenge, with 11 million sepsis-related deaths reported in 2017, representing approximately 20% of all deaths internationally (1). In accordance with the Sepsis-3 definition, sepsis is a life-threatening organ dysfunction caused by a dysregulated host response to bacterial, viral, or fungal infection (2). The hallmarks of sepsis include disturbed hemostasis and a dysregulated systemic inflammatory state (3, 4). Gram-positive bacteria, such as Staphylococcus aureus and Streptococcus pneumoniae, are a common cause of sepsis and account for about 40 to 50% of all cases (5–7). Streptococcus pyogenes can cause diseases ranging from mild throat and skin infections to invasive infections resulting in more than 500,000 deaths annually worldwide as a result of sepsis and autoimmune rheumatic fever (8). The bacteria have developed multiple mechanisms to colonize, disseminate, and evade the host immune system (9, 10). One important streptococcal virulence factor is the cell wall-anchored M protein that covers the bacterial surface. M protein is encoded by the *emm* gene, and serotyping of S. pyogenes is based on *emm* amino acid sequence variations in the N-terminal region. Another classification system, which is based on A, B, C,and D domain arrangements of the M proteins, can be used to assign serotypes into groups; patterns A-C, D, and E, which are associated with relatively distinct tissue tropisms of skin and throat or as generalists that can occupy both niches (11). The *emm* pattern A-C contains all A, B, C, and D domains, the *emm* pattern D contains B, C, and D domains, and the *emm* pattern E only contains the C and D domains (12, 13). There are more than 200 different *emm* serotypes; however, fewer than 10 serotypes are predominant in clinically significant invasive streptococcal infections (14, 15). The four serotypes *emm*1, *emm*28, *emm*3, and *emm*89 account for about 50 to 70% of all invasive S. pyogenes infections in Europe and North America, with the *emm*1 serotype being the most prevalent (14, 16). These four serotypes represent the distinct patterns of A to C or E, respectively, implying that distinct tissue tropisms may not be associated with invasive infection. M proteins use distinct binding domains to interact with several host plasma proteins, and the binding repertoire of the *emm*1 serotype (M1 protein) includes fibrinogen, albumin, the Fc-domain of IgG, and complement regulatory proteins, contributing to evasion of the complement system and phagocytosis (17–19). In addition, the M1 protein (*emm*1 serotype) can also be cleaved from the bacterial surface, both by the streptococcal cysteine protease SpeB (20) and by host-derived proteases such as neutrophil elastase (21). The ability to shed a dominant surface protein during an infection may have important implications for the functional effects of this virulence factor during distinct phases of pathogenesis. The released M1 protein binds fibrinogen within a specific concentration range and mediates activation of neutrophils (21, 22). The released M1 protein exhibits proinflammatory effects by activation of monocytes (23) and T cells (24). We have previously demonstrated that the M1 protein can activate platelets (25, 26) and stimulate platelet-leukocyte complex formation (27). We have also shown that platelet activation and neutrophil activation mediated by M1 protein are dependent on fibrinogen and specific anti-M1 protein IgG engaging with both the Fc and the fibrinogen receptors on the platelet surface (21, 22, 26). Platelets are the main regulators of hemostasis, and they also contribute to immune responses as rapidly responding sentinel cells in the blood (28, 29). Platelets use distinct repertoires of multiple surface receptors to directly recognize and respond to pathogenic bacteria, bacterium-derived cell wall components (lipopolysaccharide [LPS]), and pathogenic viruses, recently reviewed by Maouia et al. (30). Fibrinogen is a predominant blood protein that responds to infection as an acute-phase reactant and mediator of inflammation, interacting with leukocytes and platelets. Some bacterial pathogens, including S. pyogenes, bind fibrinogen to subvert fibrinogen-mediated host antimicrobial function or facilitate invasion within the host (31). The ability of the M proteins to acquire plasma fibrinogen is not conserved for all serotypes and may be associated with distinct serotype patterns (12, 13). In this study, we assess the importance of fibrinogen acquisition for platelet and platelet-dependent neutrophil and monocyte activation mediated by invasive serotypes of M protein from two serotype patterns, the M1, M3, and M5 proteins from pattern A-C and the M28, M49, and M89 proteins from pattern E.

## RESULTS

### M proteins exhibit serotype-specific acquisition of fibrinogen from human plasma.

**FIG 1:**
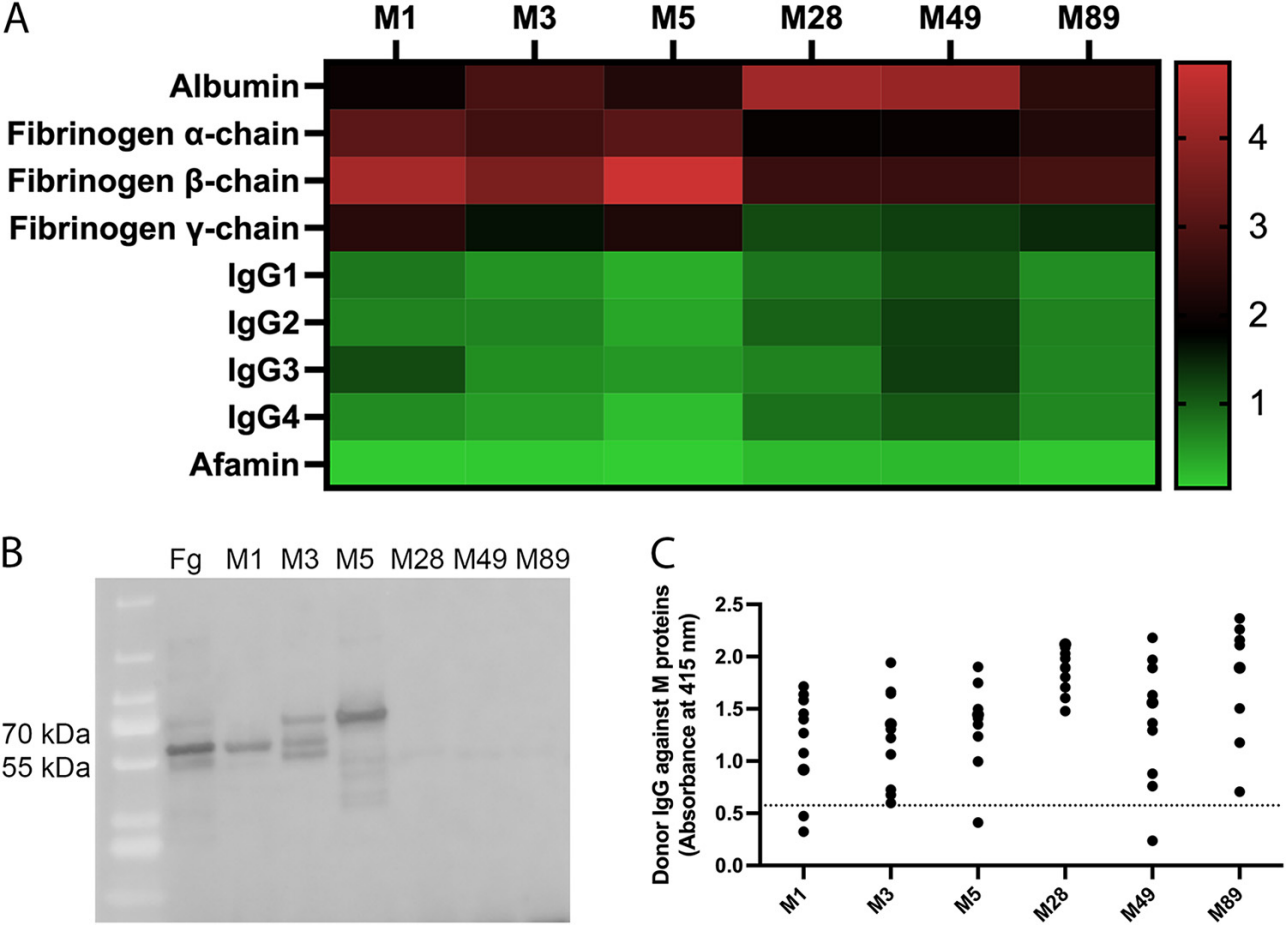
M proteins exhibit serotype-specific acquisition of fibrinogen from human plasma. (A) Fibrinogen and IgG binding to the distinct serotypes of purified M proteins; M1, M3, M5, M28, M49, and M89 protein were investigated using quantitative mass spectrometry of plasma from five healthy donors. The data are shown as the median summed total ion current (TIC) intensity for the interaction between the M proteins and plasma proteins, fibrinogen α-, β- and γ-chains, and IgG subclasses 1, 2, 3, and 4. Albumin is shown as a comparative control for interaction with M proteins. Afamin is shown as a negative control for interaction with M proteins. (B) Fibrinogen (Fb) binding to the purified M proteins was verified using immunoblotting, and fibrinogen was used as a positive control for detection. (C) ELISA was used to investigate the plasma levels of anti-M protein-specific IgG in plasma from 10 healthy donors. The absorbance at 415 nm is presented, and the dotted line represents the PBS background signal.

Plasma protein interactomes of the purified proteins, M1, M3, M5, M28, M49, and M89 proteins, were investigated. The M proteins were added to plasma from five healthy donors, and the intensities of the plasma proteins pulled down in association with the M proteins were compared using quantitative mass spectrometry. This demonstrated that fibrinogen binding only occurred for A-C pattern M protein serotypes. Fibrinogen interaction was greater with M1, M3, and M5 proteins than with M28, M49, and M89 proteins (Kruskal-Wallis test, *P* = 0.0164 [α-chain], *P* = 0.0056 [β-chain], and *P* = 0.0018 [γ-chain]) (Fig. 1A). The serotype-specific interaction with fibrinogen was confirmed using immunoblotting, where M1, M3, and M5 proteins bound fibrinogen, but M28, M49, and M89 proteins failed to acquire detectable levels of fibrinogen (Fig. 1B). In contrast, all M proteins investigated bound relatively equivalent levels of all four subclasses of IgG and albumin from plasma (Fig. 1A). Afamin, a vitamin E-binding plasma protein, had the lowest intensity of M protein interaction in this study for all serotypes (Fig. 1A). The interaction with IgG was further investigated using enzyme-linked immunosorbent assay (ELISA) to distinguish Fab-bound from Fc-bound IgG in plasma from 10 healthy donors. All M proteins acquired Fab-bound IgG from plasma. The majority of the donors had IgG against all of the M proteins, indicating previous exposure to these serotypes or generation of cross-reactive antibodies to multiple serotypes (Fig. 1C).

### Platelet activation is mediated by distinct M protein serotypes.

**FIG 2:**
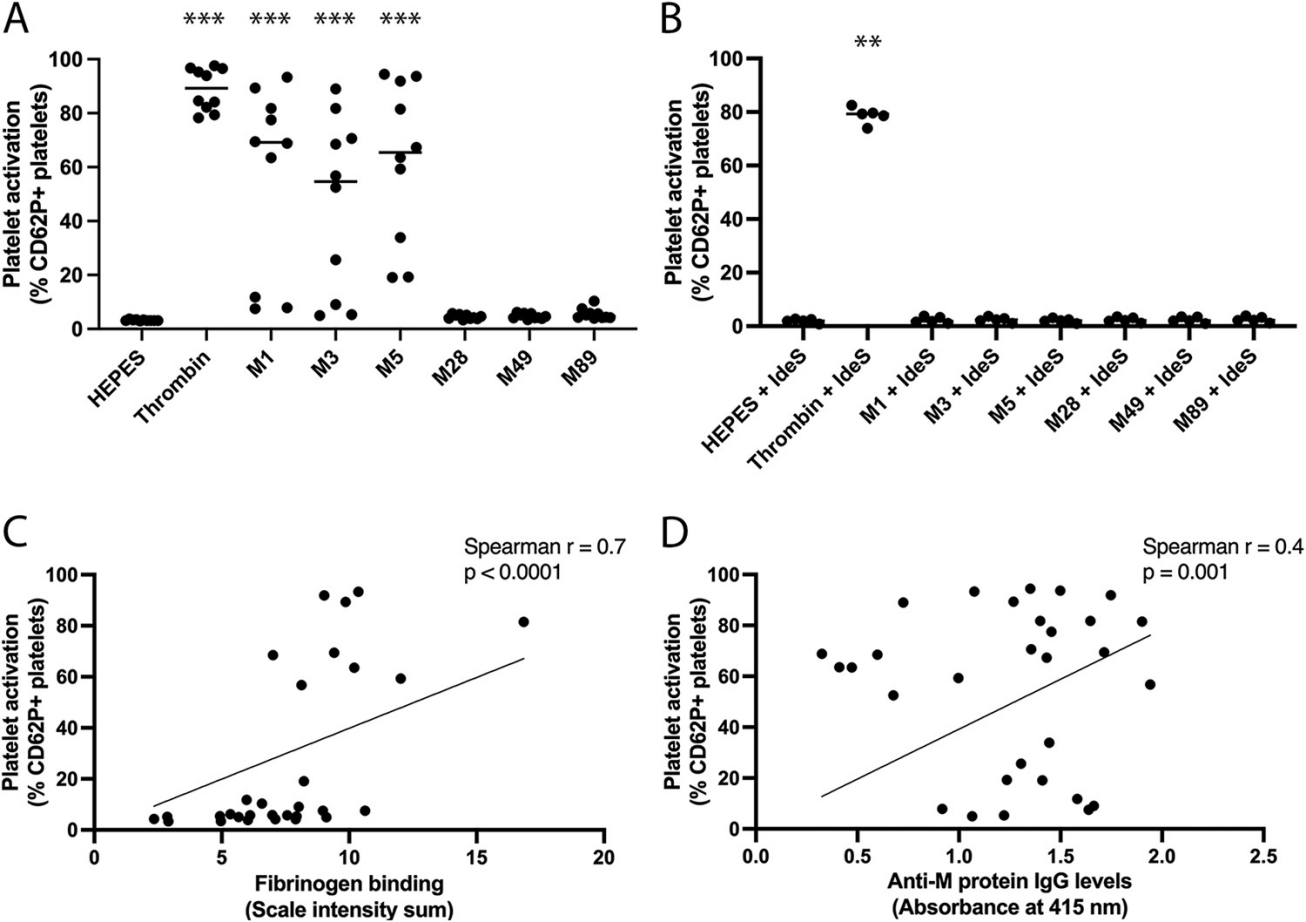
Platelet activation is mediated by distinct M protein serotypes. (A and B) Platelet activation by the distinct serotypes of purified M proteins; M1, M3, M5, M28, M49, and M89 protein were investigated using flow cytometry of platelet-rich plasma (PRP) from 10 healthy donors (A) or IdeS-treated PRP from five healthy donors (B). Platelet activation is presented as the percentage of platelets positive for CD62P. Thrombin was used as a positive control for platelet activation, and HEPES buffer was used as a control for background platelet activation. ***, *P* < 0.001; **, *P* < 0.01; Mann-Whitney test (panels A and B). (C and D) The potential correlations between M protein-mediated platelet activation and M protein interaction with fibrinogen (C) or anti-M protein IgG levels (D) were investigated using Spearman correlation tests.

Platelet activation was investigated using flow cytometry of platelet-rich plasma from 10 healthy donors. M1, M3, and M5 protein mediated significant platelet activation, at equivalent levels to the platelet agonist thrombin. Medians of 69%, 55%, and 65% of the platelet population became activated, respectively, compared to the background level of only 3% (Fig. 2A). A donor-dependent variation was observed for M protein-mediated platelet activation, with a low level of platelet activation in some donors, whereas thrombin-mediated platelet activation was homogenous. M28, M49, and M89 protein failed to mediate platelet activation above background levels in all donors. Platelet activation initiated by M1, M3, and M5 protein was abolished after pretreatment with the IgG-cleaving enzyme IdeS, whereas platelet activation by thrombin was unaffected by IdeS pretreatment (Fig. 2B). This demonstrates that M protein-induced platelet activation is dependent on intact IgG. Furthermore, Spearman correlation tests showed a statistically significant correlation between M protein-mediated platelet activation and fibrinogen binding to M protein (Fig. 2C) or specific IgG (Fig. 2D). A higher spearman *r* value was observed for fibrinogen than for IgG. Collectively, this indicates that M protein-mediated platelet activation requires both binding of fibrinogen and specific IgG to M protein.

### Platelet granule release and platelet aggregation is mediated by distinct M protein serotypes.

**FIG 3:**
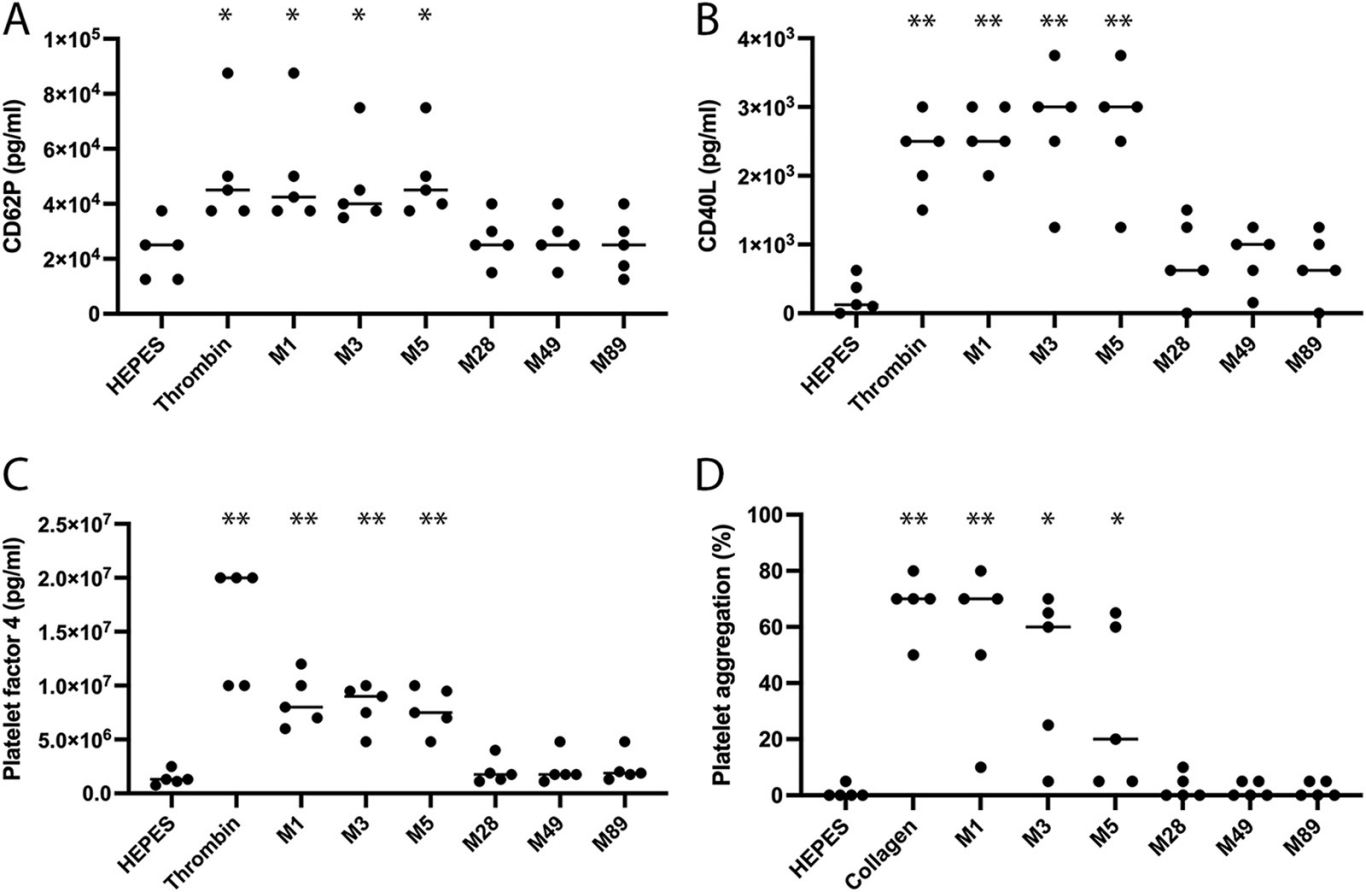
Platelet granule release and platelet aggregation are mediated by distinct M protein serotypes. (A to C) Platelet granule release mediated by the distinct serotypes of purified M proteins; M1, M3, M5, M28, M49, and M89 protein were investigated using commercial CD62P, CD40L, and platelet factor 4 ELISA kits with platelet supernatants from five healthy donors (Fig. 3A to C). Thrombin was used as a positive control for platelet granule release, and HEPES buffer was used as a control for background platelet granule release. (D) Platelet aggregation mediated by the distinct serotypes of purified M proteins; M1, M3, M5, M28, M49, and M89 protein were investigated using platelet aggregometry with PRP from five healthy donors. Collagen was used as a positive control for platelet aggregation, and HEPES buffer was used as a control for background platelet aggregation. **, *P* < 0.01; *, *P* < 0.05; Mann-Whitney test.

Platelet granule release was investigated using commercially available ELISA kits to measure CD62P, CD40L, and platelet factor 4 in the supernatant of activated platelets from five healthy donors. M1, M3, and M5 protein mediated significant release of CD62P (Fig. 3A), CD40L (Fig. 3B), and platelet factor 4 (Fig. 3C), at equivalent levels to the platelet agonist thrombin. M28, M49, and M89 protein failed to mediate platelet granule release above background levels. Furthermore, platelet aggregation upon stimulation with the different M proteins was investigated. M1, M3, and M5 protein mediated significant platelet aggregation (Fig. 3D), at equivalent levels to the platelet agonist collagen, with a lag time of 4 to 7 min. A donor-dependent variation was observed for M protein-mediated platelet aggregation, with a low level of platelet aggregation occurring in some donors, whereas collagen-mediated platelet aggregation was homogenous. M28, M49, and M89 protein failed to mediate platelet aggregation above background levels in all donors.

### Platelet-neutrophil and platelet-monocyte complex formation occurs in response to distinct M protein serotypes.

**FIG 4:**
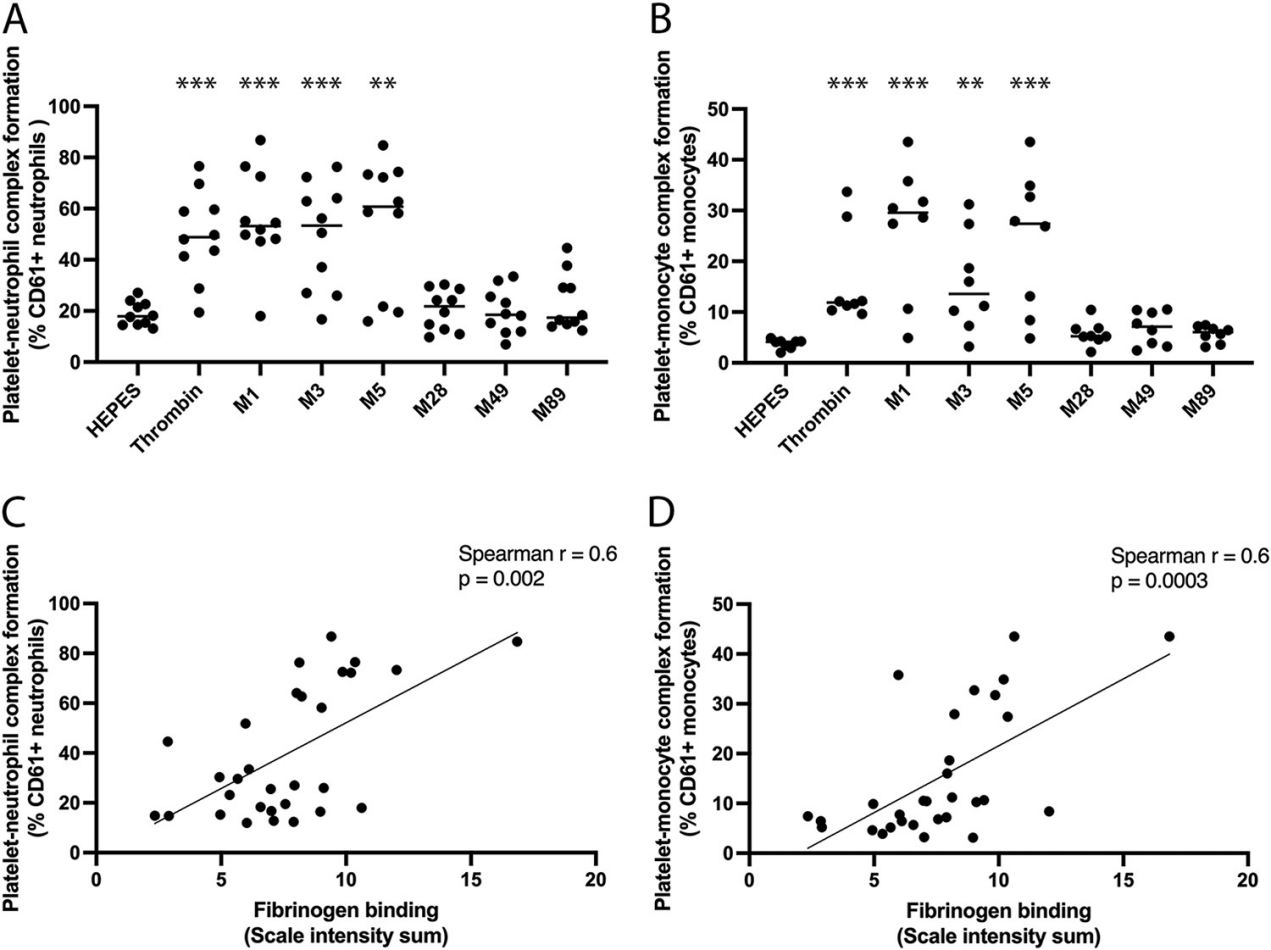
Platelet-neutrophil and platelet-monocyte complex formation occur in response to distinct M protein serotypes. (A and B) Flow cytometry was used to assess platelet-neutrophil complex (PNC) formation (A) and platelet-monocyte complex (PMC) formation (B) by the distinct serotypes of purified M proteins; M1, M3, M5, M28, M49, and M89 protein in blood from 10 healthy donors. Data are presented as the percentage of neutrophils (A) and monocytes (B) that are CD61 positive (platelet associated). Thrombin is used as a positive control for platelet activation and platelet-leukocyte complex formation (panels A and B). HEPES buffer is used as a control for background PNC and PMC formation. **, *P* < 0.01; ***, *P* < 0.001. (C and D) The potential correlations between M protein interaction with fibrinogen- and M protein-mediated PNC formation (C) or PMC formation (D) were investigated using Spearman correlation tests.

Activated platelets can bind to leukocytes; therefore, platelet-neutrophil and platelet-monocyte complex formation were investigated using flow cytometry of whole blood from 10 healthy donors. M1, M3, and M5 protein generated platelet-neutrophil complex formation with a median level of 53%, 53%, and 61% of the neutrophil population, compared to the background level of 18% (Fig. 4A). Thrombin also mediated platelet-neutrophil complex formation, with a median of 49% platelet-positive neutrophils (Fig. 4A). Furthermore, M1, M3, and M5 protein generated platelet-monocyte complex formation, although to a lesser degree, with a median of 30%, 17%, and 26% of the monocyte population, compared to the background level of 4% (Fig. 4B). Thrombin mediated platelet-monocyte complex formation with a median of 49% platelet-positive monocytes (Fig. 4B). M protein-mediated platelet-neutrophil and platelet-monocyte complex formation both correlated with the ability of M protein to bind fibrinogen (Fig. 4C and D). Some interindividual variation was observed among donors for both thrombin- and M protein-mediated complex formation. M28, M49, and M89 protein failed to generate platelet-neutrophil or platelet-monocyte complex formation.

**FIG 5:**
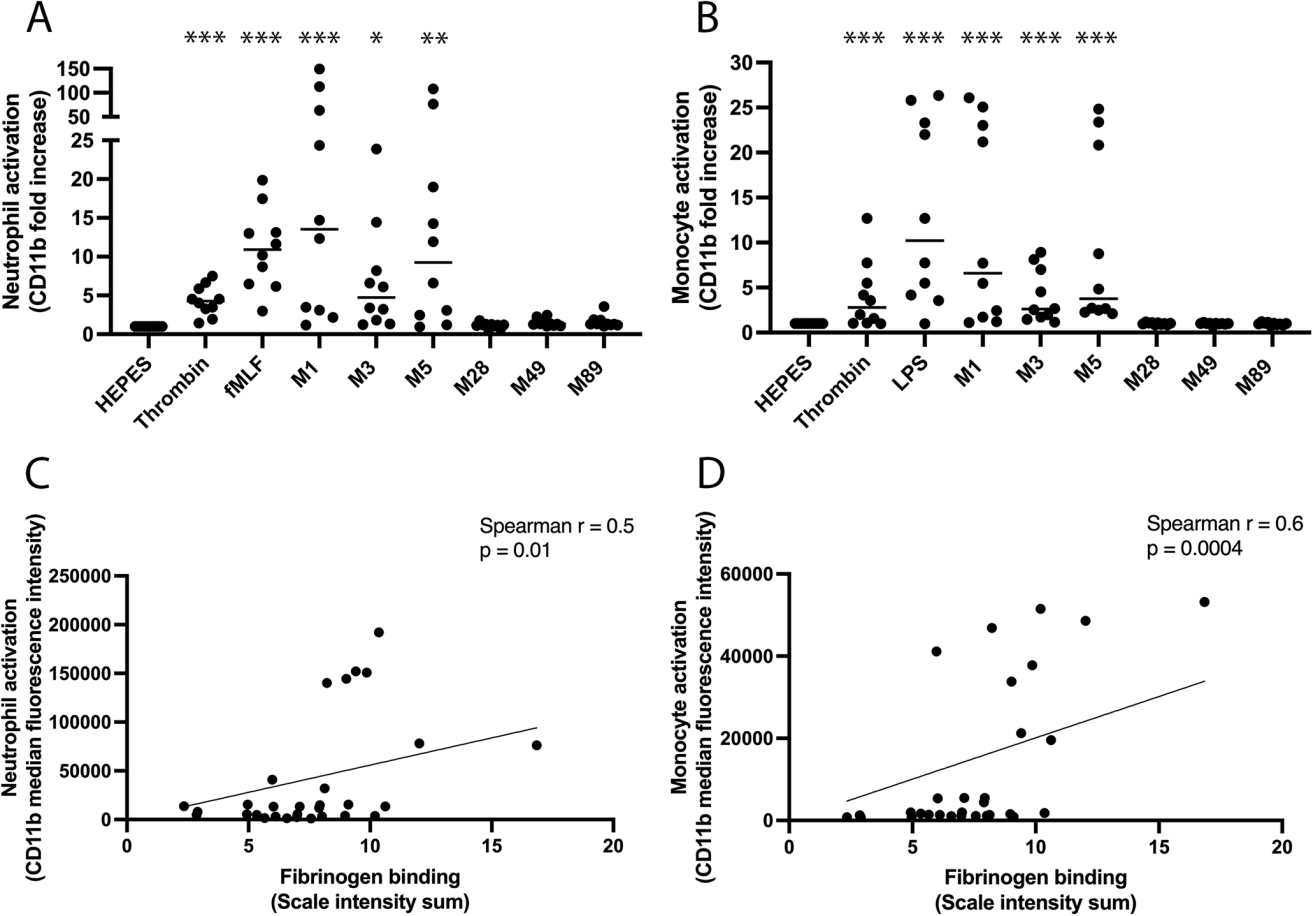
Neutrophil and monocyte activation occur in response to distinct M protein serotypes. (A and B) Flow cytometry was used to assess neutrophil activation (A) and monocyte activation (B) by the distinct serotypes of purified M proteins; M1, M3, M5, M28, M49, and M89 protein in blood from 10 healthy donors. The data are presented as the fold increase of CD11b median fluorescence intensity compared to baseline levels in HEPES buffer controls. Thrombin, fMLF, and LPS were used as positive controls for platelet, neutrophil, and monocyte activation, respectively (panels A and B). *, *P* < 0.05; **, *P* < 0.01; ***, *P* < 0.001. (C and D) The potential correlations between M protein interaction with fibrinogen- and M protein-mediated neutrophil activation (C) or monocyte activation (D) were investigated using Spearman correlation tests.

**FIG 6:**
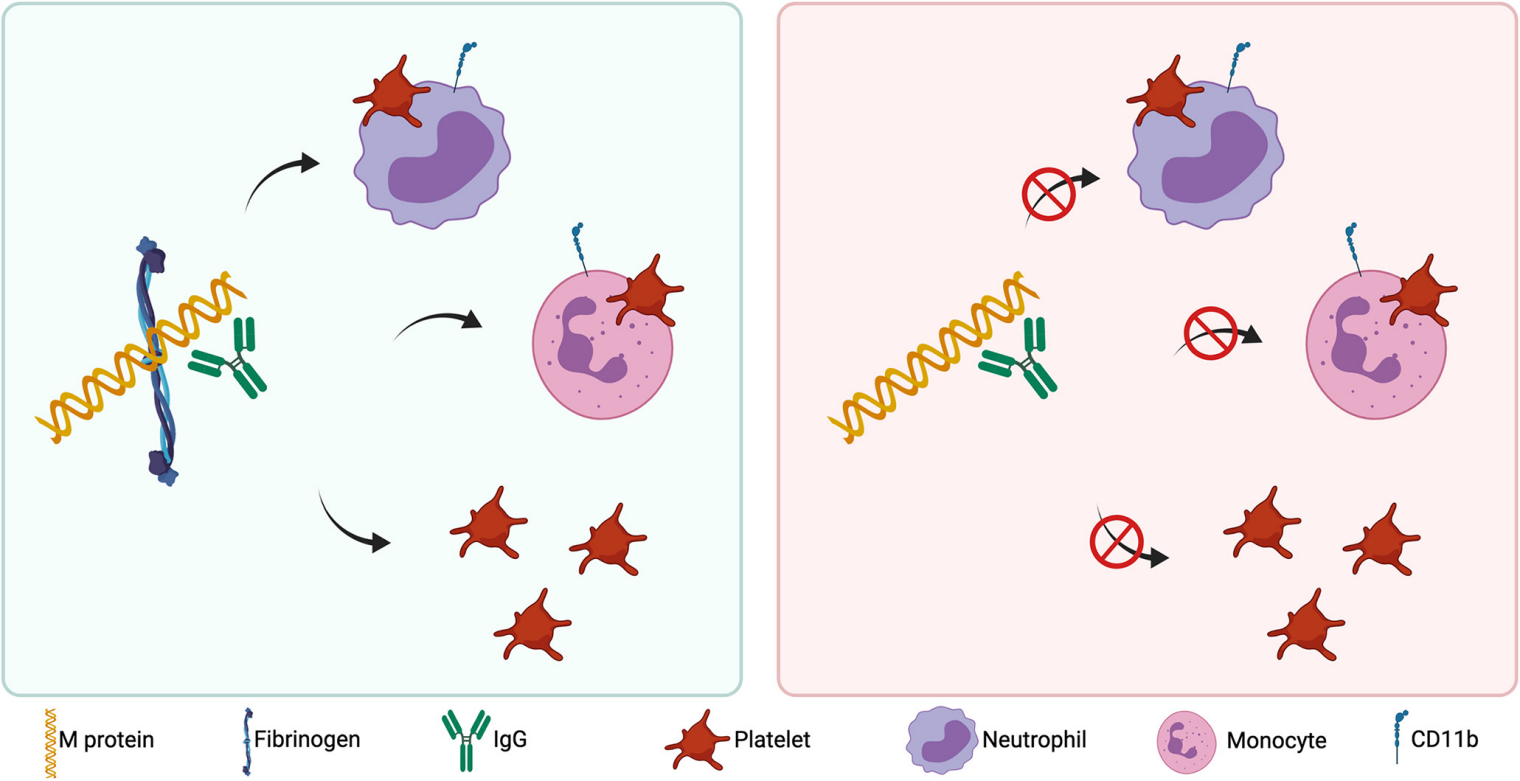
Distinct serotypes of streptococcal M proteins mediate fibrinogen-dependent platelet activation, platelet-leukocyte complex formation, and leukocyte activation. M protein serotypes that interact with fibrinogen and anti-M protein IgG mediate platelet activation, release of proinflammatory platelet granule proteins, platelet-neutrophil complex formation, platelet-monocyte complex formation, and leukocyte activation (green box). M protein serotypes that do not interact with fibrinogen do not mediate platelet-dependent inflammatory responses (red box).

In parallel analyses, neutrophil and monocyte activation were investigated. M1, M3, and M5 protein mediated significant upregulation of CD11b in the neutrophil population with fold increases of 14, 5, and 9, respectively, in the median fluorescence intensity (MFI) above background (Fig. 5A). The neutrophil agonist, *N*-formylmethionine-leucyl-phenylalanine (fMLF), and the platelet agonist, thrombin, also mediated neutrophil activation, with a more potent activation observed for fMLF (Fig. 5A). Furthermore, M1, M3, and M5 protein mediated significant upregulation of CD11b in the monocyte population with fold increases of 7, 3, and 4, respectively, in the MFI above background (Fig. 5B). The monocyte agonist, LPS, and the platelet agonist, thrombin, also mediated monocyte activation, with a more potent activation observed for LPS (Fig. 5B). Equivalent levels of interindividual donor variation were observed for all agonists tested. M28, M49, and M89 protein failed to mediate neutrophil or monocyte activation under these experimental conditions. M protein-mediated neutrophil and monocyte activation both correlated with the ability of M protein to bind fibrinogen (Fig. 5C and D). Collectively, this indicates that distinct serotypes of M protein bind fibrinogen and mediate fibrinogen-dependent platelet activation, platelet-leukocyte complex formation, and leukocyte activation as illustrated in Fig. 6.

## DISCUSSION

The transition from a local infection to an invasive infection exposes S. pyogenes to different environments and is associated with distinct serotypes. In the bloodstream the bacteria encounter an abundant repertoire of plasma proteins and circulating immune cells, with which the bacteria exhibit serotype-dependent interactions. In this study, we demonstrate that only M protein released from distinct streptococcal serotypes (M1, M3, and M5 proteins) bind plasma fibrinogen and Fab-bound IgG to mediate rapid platelet activation, complex formation with neutrophils and monocytes, and activation of these classical drivers of inflammation. The M28, M49, and M89 proteins failed to engage fibrinogen or mediate platelet-dependent inflammation. The serotypes of M protein investigated in this study are all associated with invasive infections (14, 16), indicating that fibrinogen binding to the M protein is not a prerequisite for invasive infection; however, proinflammatory strategies differ between serotypes. Our findings shed light on the importance of M protein binding to fibrinogen and platelets in the immune response to S. pyogenes infection.

We demonstrate that M protein-mediated platelet activation is IgG-Fc receptor dependent, since IgG cleavage by IdeS treatment abolishes platelet activation. This is in line with previous findings for M1 protein (25, 26) and is now expanded to additional serotypes for which M protein-specific plasma IgG levels correlate with platelet activation. Interestingly, the majority of healthy individuals recruited in our study exhibited anti-M protein-specific plasma IgG against multiple serotypes, indicating previous exposure to S. pyogenes of the same serotype or generation of cross-reactive anti-M protein IgG to conserved regions on the M protein. Our findings demonstrate that IgG binding alone is not sufficient for platelet activation to occur, as only released M proteins that acquire fibrinogen mediate platelet activation despite the presence of IgG against all serotypes. The inability of fibrinogen-binding M proteins to activate platelets in certain donors contributes to the variation observed, which impacts upon the correlations observed between platelet activation, M protein fibrinogen binding, and levels of M protein-specific IgG. The ability of the B-repeats from M1, M3, and M5 proteins to bind the D domain of fibrinogen has previously been established (19, 32–35). An *emm*-type classification system based on closely related M proteins that share binding and structural properties (36) predicts that only M proteins containing the B-repeats bind fibrinogen. M28, M49, and M89 belong to pattern E, which lacks B-repeat domains required for direct binding to fibrinogen. The lack of B-repeat domains likely explains the inability of these released M protein serotypes to bind fibrinogen under our experimental conditions. An essential role for the fibrinogen binding domain of the M1 protein and engagement of the platelet fibrinogen receptor (GPIIb/IIIa) has previously been demonstrated for platelet activation mediated by M1 protein (25). Furthermore, other clinically relevant bacterial pathogens, including Streptococcus pyogenes and Staphylococcus aureus, require both fibrinogen binding and IgG opsonization to mediate platelet activation (37). The platelet fibrinogen receptor (GPIIb/IIIa) can also amplify the signal from the platelet IgG receptor (FcγRIIA) in response to bacteria (38).

Fibrinogen is an acute-phase protein that participates in coagulation, inflammation, and tissue repair during infection; however, many pathogenic bacteria can manipulate these fibrinogen-dependent pathways (31). We have now determined that serotype-specific interactions with fibrinogen occur for M proteins released from S. pyogenes. In particular, the inability of M28, M49, and M89 to engage fibrinogen compromises the ability of these proteins to mediate fibrinogen-dependent proinflammatory effects in the blood. The serotypes that do not bind fibrinogen via the M protein might acquire fibrinogen to the surface via another surface protein in the M-related protein family (12). Recent work has shown that the *emm*28, -49, and -89 bacterial serotypes from pattern E also bind significantly less fibrinogen to the bacterial surface than the *emm*1, -3, and -5 serotypes (39) from pattern A-C. Distinct human plasma-interactome networks were observed for the serotypes from pattern A-C that acquired mainly fibrinogen and complement proteins, while pattern E serotypes acquired mainly apolipoproteins, immunoglobulins, and coagulation proteins. The ability to bind coagulation proteins provides a mechanism for the bacteria to indirectly interact with fibrinogen and fibrinogen-dependent coagulation pathways. Acquisition of plasminogen to the bacterial surface mediates fibrinolysis, which is essential for multiple aspects of invasive S. pyogenes infection (40). Collectively, we have shown that direct binding of fibrinogen is more predominantly associated with distinct serotypes of both the bacteria and the released bacterial proteins, which highlights the importance of fibrinogen acquisition and manipulation for distinct serotype-specific manifestations of S. pyogenes infection.

Fibrinogen binding to the surface of S. pyogenes has previously been investigated as an immune manipulation strategy for the bacteria. Binding of fibrinogen to the M5 protein on the bacterial surface has been linked to phagocytosis resistance (34) and inhibition of complement deposition (41). The M5 protein, as well as the fibrinogen-binding B-repeats of the M5 protein, activates T cells and macrophages in immunized mice (42). The M5 protein has recently been shown to be essential for manipulation of type 1 interferon (IFN)-dependent production of the anti-inflammatory cytokine interleukin-10 (IL-10) from infected macrophages, although this was independent of fibrinogen binding to the B-repeats of M5 protein (43). Our findings provide another potential immune manipulation strategy for fibrinogen acquisition. An important component of our study is that the released form of M protein binds fibrinogen; therefore, this interaction will occur at a distance from the bacterial surface, and the functional consequences for the pathogen may be different. The ability to activate platelets at a distance from the bacterial surface could be a novel immune evasion strategy to avoid direct contact with antimicrobial substances released by activated platelets or entrapment of bacteria in a platelet aggregate (44).

Our findings also describe a novel proinflammatory effect of fibrinogen acquisition by mediating platelet activation. Only the M proteins that bind fibrinogen mediate platelet activation, and fibrinogen binding correlates with platelet activation. Platelet activation and aggregation by the bacteria may contribute to platelet depletion during invasive S. pyogenes infection. Thrombocytopenia, a low platelet count, occurs in sepsis and correlates with pathogenesis and severity of disease (45). We have previously reported that platelet activation, platelet-neutrophil complex formation, and thrombocytopenia occur in a mouse model of S. pyogenes serotype *emm*1 sepsis (46, 47). In the present study, we confirm that M protein-mediated platelet-leukocyte complex formation is fibrinogen-dependent (25) and demonstrate that only distinct serotypes exhibit this proinflammatory ability—platelet activation and platelet-neutrophil and platelet-monocyte complex formation. We conclude that platelet-neutrophil and platelet-monocyte complex formation are dependent on platelet activation since the platelet-specific agonist thrombin results in platelet-neutrophil and platelet-monocyte complex formation (Fig. 4), in the absence of leukocyte activation (Fig. 5). Our findings show that platelets activated by bacterial proteins can release immunomodulatory granule proteins (CD40L) and engage and activate leukocytes, further highlighting the proinflammatory role of platelets during infection and sepsis. Furthermore, we demonstrate novel serotype-specific consequences of fibrinogen acquisition and platelet activation during the immune response to S. pyogenes infection.

## MATERIALS AND METHODS

### Blood collection and preparation.

Informed consent was obtained prior to blood collection from 10 healthy donors (donors 1 to 10; see Fig. S1 in the supplemental material) into 0.1 M Na_3_ citrate as an anticoagulant. Ethical approval was obtained from the local ethics committee (approval 2015/801), and the study was conducted according to the Declaration of Helsinki. The blood was centrifuged at 150 × *g* for 15 min to obtain platelet-rich plasma (PRP) and at 2,000 × *g* for 10 min to obtain platelet-poor plasma (PPP or plasma).

### Cloning, expression, and purification of recombinant proteins.

The cloning, expression, and purification of the recombinant proteins used in this study have been described previously (39). For this study, the proteins were further purified using StrepTactin Sepharose (Sigma-Aldrich), washed with phosphate-buffered saline (PBS), and eluted with 5 mM biotin in PBS (Sigma-Aldrich). SDS-PAGE was performed as a quality control of the protein purity.

### Sample preparation for mass spectrometry.

Formation of protein complexes in plasma on addition of M proteins was performed as previously described (22). M1, M3, M5, M28, M49, or M89 protein (5 μg/mL) was incubated with human plasma diluted 1:10 in Dulbecco’s PBS (D-PBS) (Sigma-Aldrich) from five healthy donors (donors 1 to 5) for 1 h at room temperature. The samples were centrifuged at 12,000 × *g* for 1 min, and the pellets were washed and resuspended in 50 μL D-PBS. These samples were prepared for data-independent acquisition mass spectrometry (DIA-MS) as previously described (39). In short, the proteins were denatured with 8 M urea and 100 mM ammonium bicarbonate (Sigma-Aldrich) at room temperature for 30 min, and the disulfide bonds were reduced using 0.5 mM Tris(2-carboxyethyl)phosphine hydrochloride (TCEP) (Sigma-Aldrich) at 37°C for 60 min and alkylated with 20 mM 2-iodoacetamide (IAA) (Sigma-Aldrich) for 30 min in the dark at room temperature. The digested samples were diluted with 100 mM ammonium bicarbonate (Sigma-Aldrich) to a urea concentration of 0.8 M and digested with 3.6 μg/μL sequencing-grade trypsin (Promega) for 18 h at 37°C. The reactions were stopped by the addition of 10% formic acid (Sigma-Aldrich) to a final pH of 3. The peptides were purified using UltraMicroSpin silica C_18_ 300 Å columns (no. SUM SS18V; The Nest Group) according to the manufacturer’s instructions. The eluted peptides were dried in a SpeedVac and reconstituted in 2% acetonitrile and 0.1% formic acid with iRT (retention time) peptides (48) for liquid chromatography tandem mass spectrometry (LC-MS/MS) analysis.

### Liquid chromatography tandem mass spectrometry (LC-MS/MS).

The peptides were analyzed using DIA-MS on a Q Exactive HFX mass spectrometer (Thermo Scientific) connected to an EASY-nLC 1200 instrument (Thermo Scientific). The peptides were separated on a Thermo EASY-Spray column (Thermo Scientific; inside diameter [i.d.] 75 μm by 50 cm, column temperature 45°C) operated at a maximum pressure of 800 bar. A linear gradient of 4 to 45% acetonitrile in aqueous 0.1% formic acid was run for 50 min. One full MS scan (resolution 60,000 for a mass range of 390 to 1210 *m/z*) was followed by 32 MS/MS full-fragmentation scans (resolution 30,000) using an isolation window of 26 *m/z* (including 0.5 *m/z* overlap between the previous and next window). The precursor ions within each isolation window were fragmented using higher-energy collisional-induced dissociation at a normalized collision energy of 30. The automatic gain control was set to 3e^6^ for MS and 1e^6^ for MS/MS. All data analyses were stored and managed using openBIS (49). Raw data files were converted to mzXML using msconvert and analyzed using OpenSWATH 2.0.1 (revision c23217e) and a previously established spectral library (39). RT was calibrated using iRT peptides (48). Peptide precursors were identified by OpenSWATH 2.0.1, and PyProphet 2.0.1 was used to control the false-discovery rate (FDR) of 1% at the peptide precursor and protein level. TRIC (50) was enabled, but realigned and requantified values were subsequently removed. The data were filtered as previously described using a log_2_-fold enrichment with a >2 cutoff and an adjusted *P* value of <0.05 using Student’s *t* test (32).

### Assessing the fibrinogen-binding properties of the M proteins.

The ability of the M proteins to bind fibrinogen was investigated using immunoblotting. M proteins (25 μg/mL) were diluted 1:5 with sample buffer (Pierce) and run on a Mini-PROTEAN TGX precast gel (Bio-Rad). Fibrinogen (25 μg/mL) (Sigma-Aldrich) was included as a positive control. The proteins were transferred to a membrane using a Trans-Blot Turbo transfer system (Bio-Rad), and the membrane was blocked with 5% bovine serum albumin (BSA) in PBS with Tween 20 (PBST) for 30 min at 37°C. After washing with PBST, the membrane was probed with human fibrinogen (250 μg/mL) for 1 h at 37°C and washed, and binding was detected using fluorescein isothiocyanate (FITC)-labeled antifibrinogen (3.8 μg/mL) (Dako; polyclonal) for 1 h at 37°C. After a final wash, the membrane was photographed in a ChemiDoc MP imaging system (Bio-Rad) using the Alexa-488 filter.

### Quantification of anti-M protein IgG in plasma.

To investigate the plasma IgG titers against the different M proteins in 10 healthy donors (donors 1 to 10), an ELISA was performed. MaxiSorp 96-well ELISA plates (Thermo Fisher Scientific) were coated with the different M proteins (2.5 μg/mL) and left overnight at 4°C. Plasma from the 10 donors was pretreated with the specific IgG-cleaving enzyme IdeS (20 μg/mL) for 30 min at room temperature to generate Fab- and Fc-fragments. SDS-PAGE of IdeS-treated plasma was performed to confirm that IgG was cleaved by the treatment. The ELISA plate was washed three times with PBST and incubated with the IdeS-treated plasma (diluted 1:10) from the 10 donors for 1 h at 37°C, as previously described (26). The plate was washed, and bound IgG Fab-fragments were detected with horseradish peroxidase (HRP)-labeled anti-Fab (1:1,000) (Abcam; polyclonal). The plate was washed and developed with an ABTS [2,2′-azinobis(3-ethylbenzthiazolinesulfonic acid)] substrate (Sigma-Aldrich) before the absorbance at 415 nm was determined.

### Flow cytometry for determination of platelet activation.

Flow cytometry was used to investigate platelet activation in response to M proteins as previously described (25–27). PRP was prepared from 10 healthy donors (donor 1–10) and diluted 2:3 in 1 mM HEPES buffer, pH 7.4, and stimulated for 10 min with the different M proteins (2.5 μg/mL). HEPES buffer alone was used to determine the background platelet activation, and the platelet agonist thrombin (1 U/mL; Triolab) was used as a positive control for platelet activation in combination with the anticoagulant peptide Gly-Pro-Arg-Pro (1.25 mg/mL; Bachem). After stimulation, the samples were incubated with anti-CD62P-PE (1:10) (BD Biosciences; clone AC1.2), to detect activated platelets, for 15 min at room temperature and protected from light. The samples were run on an Accuri C6 Plus flow cytometer (BD Biosciences), and the data were analyzed using C6 Plus software. Platelets were gated based on size and granularity in logarithmic mode, and the CD62P intensity for the gated population was analyzed in histograms (Fig. S2). In order to investigate the role of IgG for M protein-mediated platelet activation, the PRP was pretreated with IdeS as described above for ELISA.

### Quantification of platelet granule release.

Platelet granule release in response to the different M proteins was investigated using ELISA. PRP was prepared from five healthy donors (donors 1 to 5) as previously described and stimulated for 120 min with the different M proteins (2.5 μg/mL). The samples were centrifuged at 2,000 × *g* for 10 min, and the CD62P, CD40L, and platelet factor 4 content in the supernatants was measured using commercially available ELISA kits (R&D Systems) according to the manufacturer’s instructions. HEPES buffer alone was used to determine the background levels of CD62P, CD40L, and platelet factor 4, and the platelet agonist thrombin (1 U/mL; Triolab) was used as a positive control for platelet activation in combination with the anticoagulant peptide Gly-Pro-Arg-Pro (1.25 mg/mL; Bachem).

### Platelet aggregation.

Platelet aggregation in response to the different M proteins was investigated using platelet aggregometry. PRP was prepared from five healthy donors (donors 1 to 5) as previously described and stimulated with the different M proteins (2.5 μg/mL) in sample wells of an aggregometer (Chrono-Log model 490). The platelet aggregation was monitored for a maximum of 25 min, or until aggregation was observed, and the percentage of platelet aggregation was determined using the software AggroLink (Chrono-Log). Plasma was used as a reference, HEPES buffer was used to determine the background platelet aggregation, and the platelet agonist collagen I (5 μg/mL; Chrono-Log) was used as a positive control.

### Flow cytometry for determination of platelet-neutrophil and platelet-monocyte complex formation.

To investigate platelet-neutrophil and platelet-monocyte complex formation and neutrophil and monocyte activation in response to the different M proteins, flow cytometry was performed. Whole blood from 10 healthy donors (donors 1 to 10) was stimulated for 10 min with M proteins (2.5 μg/mL). HEPES buffer was used to determine the background activation. The leukocyte agonist *N*-formylmethionine-leucyl-phenylalanine (fMLF) (1 μM; Sigma-Aldrich) was used as a positive control for neutrophil activation, lipopolysaccharide (LPS) Escherichia coli O111:B4 (1 μg/mL) (EMD Millipore Corp.) was used as a positive control for monocyte activation, and thrombin (1 U/mL) was used as a positive control for platelet activation in combination with the anticoagulant peptide Gly-Pro-Arg-Pro (1.25 mg/mL). After stimulation, the samples were incubated for 15 min protected from light with anti-CD61-PE (1:10) (BD Biosciences; clone VI-PL2) to detect platelets associated with leucocytes and anti-CD11b-PerCP (1:10) (BD Biosciences; clone ICRF44) to determine neutrophil and monocyte activation. The samples were run on an Accuri C6 Plus flow cytometer (BD Biosciences), and the data were analyzed using C6 Plus software. The neutrophils were gated based on size and granularity. After excluding the neutrophils, the monocytes were gated based on CD11b and granularity. The CD61 and CD11b intensity within the gates were analyzed in histograms (Fig. S3).

### Statistical analyses.

A Mann-Whitney U test was chosen to compare sample distribution in two groups for nonparametric data generated in flow cytometry, where the different M proteins were compared with the background in the presence of HEPES buffer alone. A Kruskal-Wallis test was chosen to compare three or more groups for nonparametric data generated in mass spectrometry, where the different M proteins were all compared with each other. A Spearman correlation test was chosen to investigate possible correlations of nonparametric flow cytometry, mass spectrometry, and anti-M1 protein IgG ELISA data. Median and individual values were used to present nonparametric data for continuous variables. Results were considered statistically significant if *P* was <0.05. Data were analyzed using Prism 9 (GraphPad Software).
