# Supplementary material for: Distinct Serotypes of Streptococcal M Proteins Mediate Fibrinogen-Dependent Platelet Activation and Proinflammatory Effects
Source: Infect Immun. 2022 Feb 17;90(2):e00462-21. doi: 10.1128/iai.00462-21 (PMC8852700; doi:10.1128/iai.00462-21)
Supplement: Supplemental file 1 — Supplemental material. Download iai.00462-21-s0001.pdf, PDF file, 0.6 MB [file iai.00462-21-s0001.pdf]

S1A

Donor	Sex	Age (years)
1	Male	40
2	Female	26
3	Female	26
4	Male	32
5	Female	32
6	Male	33
7	Female	34
8	Male	30
9	Female	37
10	Male	38

S1B

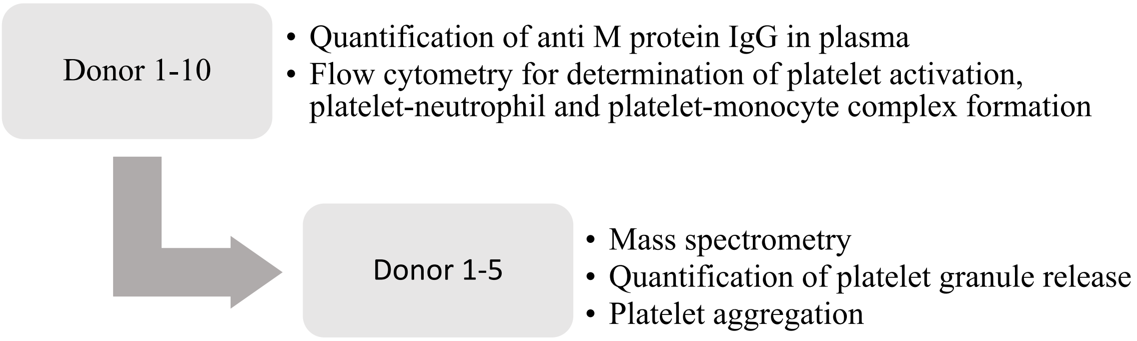

Supplementary figure 1. *Donor information.* Sex and age of the ten donors who participated in this study (Supplementary figure 1A), and description of the donors included in the different experiments (Supplementary figure 1B).

S2A S2B

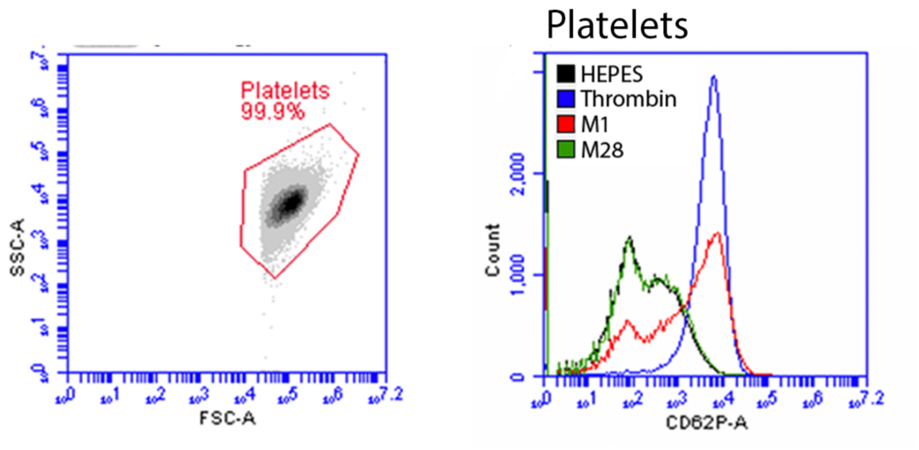

Supplementary figure 2. *Gating strategies for flow cytometry of platelet-rich plasma.* Platelets were gated based on size (FSC-A) and granularity (SSC-A) with logarithmic settings (Supplementary figure 2A), and the CD62P intensity for the gated population was analysed in histograms to determine platelet activation (Supplementary figure 2B). A representative gating strategy in response to HEPES buffer, thrombin, M1 protein and M28 protein from one experiment is shown.

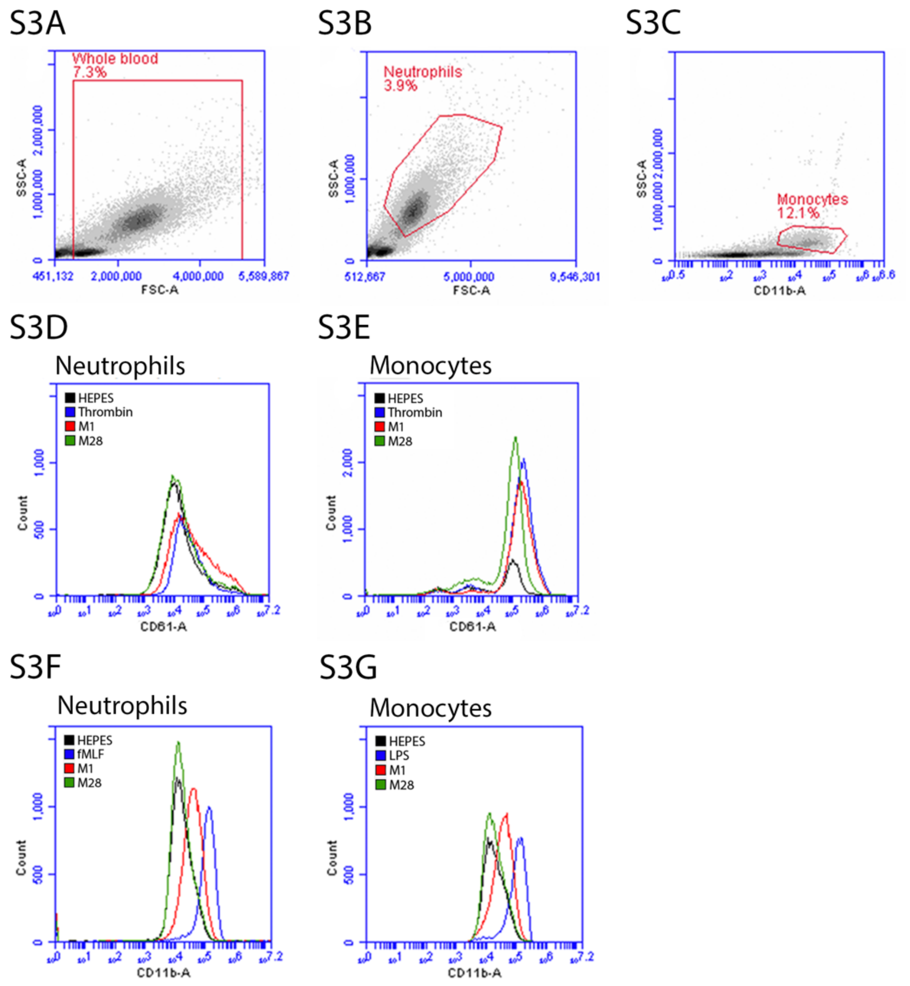

Supplementary figure 3. *Gating strategies for flow cytometry of whole blood.* The leukocyte population was gated (Supplementary figure 3A) and the granulocytes/neutrophils were gated based on size (FSC-A) and granularity (SSC-A) (Supplementary figure 3B). After excluding the neutrophils, the monocytes were gated based on CD11b and granularity (Supplementary figure 3C). The CD61 intensity within the neutrophil and monocyte gates were analysed in histograms to determine platelet-neutrophil (Supplementary figure 3D) and platelet-monocyte (Supplementary figure 3E) complex formation. The CD11b intensity within the neutrophil and monocyte gates were analysed in histograms to determine neutrophil (Supplementary figure 3F) and monocyte (Supplementary figure 3G) activation. A representative gating strategy with HEPES buffer, thrombin, fMLF or LPS, M1 protein and M28 protein from one experiment is shown.
